# Effect of Seed Traits and Waterbird Species on the Dispersal Effectiveness of Wetland Plants

**DOI:** 10.3390/biology11050629

**Published:** 2022-04-20

**Authors:** Shenghong Nie, Lizhi Zhou, Wenbin Xu

**Affiliations:** 1 School of Resources and Environmental Engineering, Anhui University, Hefei 230601, China; nieshenghong1994@163.com; 2 Anhui Shengjin Lake Wetland Ecology National Long-Term Scientific Research Base, Chizhou 247230, China; xwb197105@163.com; 3 Anhui Province Key Laboratory of Wetland Ecosystem Protection and Restoration, Anhui University, Hefei 230601, China; 4 Management Bureau of Anhui Shengjin Lake National Nature Reserve, Chizhou 247210, China

**Keywords:** endozoochory, seed dispersal, dispersal effectiveness, seed traits, dabbling duck, wetland plants

## Abstract

**Simple Summary:**

Quantifying the effects of seed traits on waterbird-mediated seed dispersal effectiveness contributes to understanding wetland plant ecology and hence can be used for improving wetland conservation and restoration, especially in the global scenario of wetland degradation and destruction. Waterbirds, especially dabbling ducks, can effectively disperse wetland plant seeds through their guts (endozoochory). Here, we experimentally quantified the effects of seed traits (length and lignin) on retention time, retrieval, and germination of surviving seeds. The results showed that the germination rate of recovered seeds was higher than the controls, suggesting that endozoochory contributes to seed germination. Local seed dispersal was more efficient, and long-distance dispersal was possible. Furthermore, smaller seeds passed through the guts faster, and there was no significant effect of disperser species on germination. We concluded that waterbird-mediated endozoochory plays an important role in wetlands.

**Abstract:**

Seed dispersal is an important ecological process in wetland ecosystems and helps maintain community structure and ecosystem biodiversity. Waterbird-mediated endozoochory is an effective and feasible dispersal mechanism for wetland plants; however, the influence of vectors and seed traits on this mechanism remains unclear. To investigate the effects of vector species and seed traits (length and lignin) on retention time, retrieval and germination of gut-surviving seeds, we fed Baikal teals (*Anas formosa*) and green-winged teals (*Anas crecca*) eight common plant seeds (*Polygonum aviculare*, *Rumex dentatus*, *Polygonum orientale*, *Vallisneria natans*, *Ranunculus polii*, *Polygonum hydropiper*, *Carex cinerascen* and *Euphrasia pectinata*) in the Shengjin Lake wetland (a Ramsar site). We collected fecal samples at intervals of 2–6 h for 36 h, and found that the percentage of recovered seeds differed significantly among teal and plant species (3%~30%); 94% of viable seeds were recovered within 12 h after feeding. Moreover, the germination rate of the recovered seeds (25%~56%) was higher than that of the control. The seed retention time was affected by seed lignin and disperser species; higher lignin made digestion difficult with higher retrieval. Smaller seeds passed through the guts but had no significant effect on recovered seeds. Seed length and disperser species showed no significant correlation with germination. These findings suggested endozoochory by dabbling ducks as an effective wetland seed dispersal mechanism.

## 1. Introduction

The dispersal of plant seeds is an essential ecological process in species distribution and is therefore a key factor in driving population spatial dynamics as well as maintaining community structure and ecosystem biodiversity on a regional scale [1,2,3,4]. Inland wetlands are often typical discrete habitats, where seed dispersal may be more important [5,6,7]. Despite the isolated characteristics and dispersal limitations of wetlands, many aquatic plants can have wide distributions, which is associated with their dispersal by migratory waterbirds [7,8,9,10]. Additionally, vector and seed traits may affect the potential for wetland plant dispersal [11].

To date, studies on seed dispersal have shown that waterbird-mediated zoochory is an indispensable mechanism for the dispersal of plants in wetland ecosystems [7,10,12]. Although individual waterbirds have a limited dispersal capacity, they have great potential owing to their enormous population size, wide distributions, and high mobility at local and global scales [12,13]. Waterbirds can disperse seeds by either attachment on the outside of their body (exozoochory) or passage through their guts (endozoochory) [14,15]. Comparative results have demonstrated that the latter is the more common and effective mode of seed dispersal [15,16], as over 97 plant species have been reported to be dispersed by waterbird endozoochory [10]. This dispersal mode allows plant seeds to overcome terrestrial barriers in order to reach isolated patches in wetlands, thereby contributing to maintaining biodiversity and promoting their recovery after disturbance, especially in the context of degradation and destruction of worldwide wetland ecosystems [13,17,18].

Dabbling ducks (Anatinae) have long been thought to be especially effective dispersers of wetland plants globally [7,14,19], as they are highly opportunistic foragers of plant seeds regardless of seed size or traits [6,20]. Ducks may even play a significant role in seed dispersal in floodplain grasslands [21]. A study on the diets of seven dabbling duck species suggested that they consume the seeds of more than 400 plant species in the Western Palearctic [9]. Although many seeds ingested by ducks could pass through their guts and germinate [10], few related studies have determined the time consumed for seeds to pass through duck’s digestive tract (retention time), the percentage of seeds that survive intact (retrievability), and the proportion of the surviving seeds germinated (germinability), and fewer controlled experiments have been performed to quantitatively compare waterbird gut-treated and untreated wetland plant seeds [5,22]. Therefore, little is known about how interactions between waterbird digestive tract treatment and seed traits affect seed dispersal effectiveness.

Waterbird-mediated seed dispersal effectiveness is largely determined by retention time, retrievability, and germinability [7,23,24]. Moreover, differences in digestive processes among waterbird species and individuals of the same species lead to different seed retention times, thereby affecting dispersal quality [25,26,27]. Experimental feeding under controlled conditions for dispersal vectors is an effective and feasible way to measure endozoochory parameters, with minimal damage to dispersal vectors [28]. Even though experimental results have repeatedly highlighted that seed retention time depends largely on seed traits [22,29,30] and digestive processes [31,32], there have been no consistent conclusions on the effect of seed traits on dispersal effectiveness. For example, smaller seeds have a higher survival rate and shorter retention time within mallard guts [29], whereas other studies showed that seed size had little impact on the dispersal potential by teals [6,28], making further research more important and necessary.

For a long time, wetlands in the middle-lower Yangtze River floodplain have been especially vital for migratory waterbirds on the East Asian-Australasian flyway; however, little attention has been paid to waterbird-mediated seed dispersal for wetland plants. To investigate the effects of interactions between seed traits (length, mass, and lignin) and disperser species on seed dispersal effectiveness, we fed two species of captive dabbling ducks (*Anas formosa* and *Anas crecca*) the seeds of eight common plant species in the Shengjin Lake wetland and quantified dispersal parameters, namely retention time, retrievability, and germination. In this study, we hypothesized that: (1) smaller seeds are more likely to survive through the digestive tract; (2) seeds with higher lignin are tough to digest and have a longer retention time, and higher retrievability and germination; (3) different disperser species have different retention time, retrievability, and germination; (4) waterbird-mediated endozoochory can promote seed germination.

## 2. Materials and Methods

### 2.1. Study Species and Seed Selection

Two different species of captive dabbling ducks, three Baikal teals (*Anas formosa)* and three green-winged teals (*Anas crecca*), were used in this feeding experiment, with both of these species common in the Shengjin Lake wetland (a Ramsar site in China; 30°15′–30°30′ N, 116°55′–117°15′ E) during the wintering period. Although both species have similar diets and habitats [9,33], they are morphologically different. We selected eight native plant seeds (*Polygonum aviculare*, *Rumex dentatus*, *Polygonum orientale*, *Vallisneria natans*, *Ranunculus polii*, *Polygonum hydropiper*, *Carex cinerascen*, and *Euphrasia pectinata*), which are common in this area [34]. Subsequently, we collected seeds of *Polygonum hydropiper*, *Polygonum orientale*, *Rumex dentatus* in September 2019 from the Shengjin Lake wetland, and the other five plant seeds were obtained from seed suppliers, without treatment. All seeds were kept dry in sealed glass bottles at room temperature and with natural light until the feeding trials the following early summer.

### 2.2. Seed Trait Measurement

To compare the effects of different seed traits on dispersal, a randomly selected subset of each seed species was oven-dried in advance at 60 °C for 48 h [28] for their sole use in measuring seed mass, length, and lignin (Table 1). A total of 10 batches of 100 dried seeds selected randomly for each species was used to measure individual seed mass with an electronic analytical balance. We considered the maximum seed length per species as a length indicator, selected 10 seeds randomly for each species, and then measured them using a digital vernier caliper (precision 0.01 mm) under a binocular microscope. We crushed 10 batches of 10 dried seeds of each plant and passed them through a 420-μm sieve, respectively, followed by determination of seed lignin content for each plant using a lignin content assay kit (AKSU010U, Beijing, China) according to manufacturer instructions. We took the mean of three consecutive times as the measurement values in each batch of all seed species.

### 2.3. Feeding Experiments

The Baikal teals and green-winged teals were selected as experimental subjects due to their marked differences in body size in order to facilitate comparison of the interaction between digestion in different species and seed traits. We performed feeding trials at the Anhui Shengjin Lake Positioning Research Station for Wetland Ecology from 13 May to 15 July 2021. Prior to the experiment, six living ducks were kept in spacious outdoor facilities and fed commercial pellets and paddy mixture, with ad libitum access to water and grit throughout the entire study period. All experimental subjects were in good condition, and we recorded the weights of these subjects as an index of their condition [35]. The feeding experiments were approved by the Institutional Animal Care and Use Committee of Anhui Zoological Society.

Previous similar feeding experiments often fed single plant seeds individually [6,29]; however, ducks have a highly mixed diet in the natural environment. Therefore, we randomly paired the seeds and fed 200 seeds (100 per plant species) to each duck at each feeding, after which they were kept individually in a cage (60 cm × 50 cm × 50 cm) for 48 h. These force-fed seeds were mixed with commercial feed, as sundried pellets make seeds easier to ingest. The bottom of each cage was covered with a plastic mat, and a removable plastic sheet was placed below that for the convenience of collecting fecal samples. The few regurgitated seeds were counted to correct the number of seeds ingested by each duck per experiment. After collecting the fecal samples, the ducks were returned to the outdoor facilities until the next experiment 5 days later.

Preliminary experiments conducted before the formal experiment revealed that there were no intact seeds or seed fragments in the feces of the Baikal teals or the green-winged teals at 36 h after seed feeding; therefore, we estimated the seed-collection time in the feces to be 36 h. Each force-feeding experiment began in the morning, and the ducks were placed in separate cages 12 h in advance in order to allow acclimatization to their environment. The feces were collected from the plastic trays every 2 h for the first 12 h and then every 4 h for the next 12 h, and finally every 6 h for the last 12 h, resulting in a total of 11 rounds of fecal-sample collection within 36 h. We checked for the presence of fecal samples that had not reached the plastic trays during each collection in order to ensure that all feces were collected. The collected feces were immediately placed in a 63-μm sieve and washed under running water in order to separate the intact seeds in a laboratory, which were then stored in tubes at 4 °C in the dark until all feeding experiments were completed, in order to start the germination of the experimental group and the control group at the same time.

We measured the body weight of each experimental subject at the end of the experimental period. Compared with the body weight before the experiment, the body weight of Baikal teals decreased slightly (mean ± SE: 451.83 ± 2.35 g vs. 445.00 ± 3.01 g; analysis of variance (ANOVA): *F*_1,22_ = 3.21, *p* = 0.087). In contrast, the body weight of the green-winged teals was lower and showed a significant decrease (321.25 ± 2.51 g vs. 312.58 ± 2.20 g; ANOVA: *F*_1,22_ = 6.74, *p* < 0.05), which may be due to stress during the trials. Eventually, all of the experimental teals were released into the wild.

### 2.4. Germination Experiment

Five groups of 10 seeds per plant species were selected randomly as the non-ingested controls and stored under the same conditions as the recovered seeds. Seeds from feeding trials and the controls were placed in Petri dishes with wet filter paper for germination in August 2020, with up to 10 seeds per dish. These Petri dishes were positioned in a phytotron with a temperature ranging from 16 °C to 28 °C and a light cycle of 16 h. Seed germination was recorded every 2 days for 6 weeks. Additionally, we replenished the water in the Petri dishes, as required, and removed any germinated seeds. After 6 weeks, the non-germinated seeds were stored in the refrigerator at 4 °C for 4 weeks, before the next germination trial was performed under the same conditions.

### 2.5. Statistical Analysis

We measured the mass, length, and lignin of the seeds of eight plant species in order to investigate whether any of these traits have a high correlation. We first analyzed the correlation between pairs of traits and found a significant correlation between seed length and mass (Spearman’s correlation: *r*-value = 0.740, *p* = 0.037), whereas the lignin content had no correlation with mass (*r*-value = −0.405, *p* = 0.319) or length (*r*-value = −0.238, *p* = 0.570). Consequently, only seed length (SL) and lignin (SLI) were selected for further analysis.

**Figure 1:**
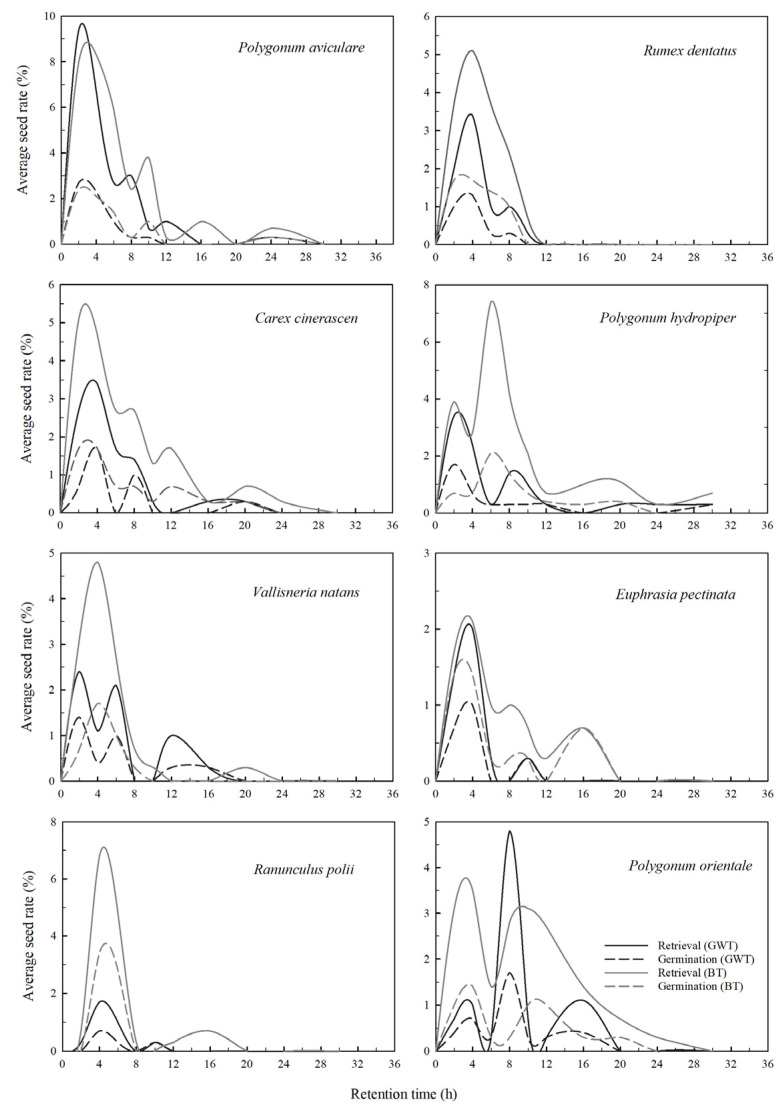
Average seed rate of recovered (smoothed solid line) and germinated (smoothed dashed line) seeds in each time interval, including eight plant species. Germination was calculated by germinated seeds per time interval and the total ingested per feeding trail. Retrieval represented the rate of seeds recovered in each time interval to the total ingested seeds during each period. GWT: green-winged teal, BT: Baikal teal.

According to the feeding and germination experimental data (Figure 1, Table 2), four main parameters related to endozoochory were included to quantify the effects of seed traits and gut processes on dispersal effectiveness: the average retention time (T_ave_) and the maximum retention time (T_max_) of recovered seeds in digestive tract, the percentage of recovered seeds (Retrieval) and the percentage of germinated seeds (Germination).

The effect of bird species (BS) and seed traits (SL, SLI), as well as their interactions, on retention time was investigated using linear mixed models (LMMs). The average and maximum retention times after log-transformation were both normally distributed as dependent variables in two separate models (LMM1a and LMM1b). Seed species (SS) and teal individual (ID) were used as random effects to reduce the effect of individual differences and species within possible equal traits in all models. The effect of BS, SL, and SLI on retrievability using generalized linear mixed models (GLMMs) with a binomial error distribution and a logit link function, as well as the total ingested seed number, was used as a binomial denominator (GLMM2). Additionally, we investigated the effect of fixed factors (BS, SL, SLI and their interaction) on germination using similar GLMMs with the same random factors; however, the total germinated seed number was used as a binomial denominator (GLMM3). Moreover, we analyzed the effect of BS and SS on germination by substituting seed traits with SS as a fixed factor; however, the results showed that the null model was the top model (results not shown).

The effects of retention time interval (RT) on the percentage of recovered and germinated seeds at different time intervals were analyzed in GLMM4a and GLMM4b with a binomial (link = logit). Because the time intervals were the same (2 h) in the first 12 h and the recovered seeds in the first 12 h accounted for 94% of the total, we analyzed only data from the first 12 h. The quadratic factor (RT^2^) and BS were also included in the models, and RT was used as a random slope and ID as a random intercept to control for individual differences at different time intervals. We compared the germination of recovered seeds from the digestive tract with the non-ingested control groups in the GLMM5 using a binomial, with SS included as a fixed factor and ID as a random factor. Moreover, the significant differences between the experiment and the control were proved by further *t*-test and 95% confidence interval analysis.

We used Akaike Information Criteria corrected (AIC_c_) to compare different possible subsets of the full models and select the best model with the lowest AIC_c_. However, if the difference (ΔAIC_c_) between other sub-models and the top was within 2, they were considered equivalent. We subsequently used model averaging to calculate the final estimates, standard errors and confidence intervals (Table 3). The *R*^2^ of the top model was used to represent the fitting effect of the model, including that marginal *R*^2^ (m*R*^2^) considered only the variance of the fixed effects, and conditional *R*^2^ (c*R*^2^) took both fixed and random effects into account. All statistical analyses were performed using R 4.1.2 [36].

## 3. Results

**Figure 2:**
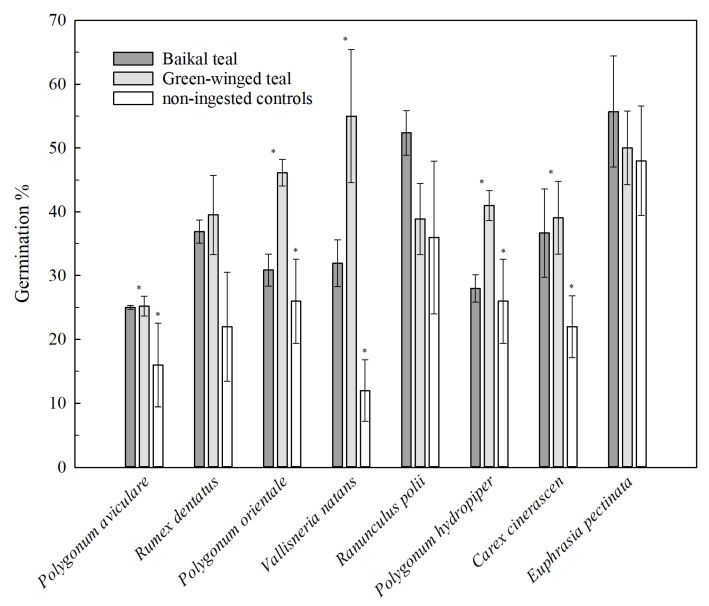
The rate of germinated seeds (±SE) ingested by Baikal teal and Green-winged teal vs. non-ingested controls. * represents the significance of the difference in germination between the experimental and control group of each seed species.

In these feeding trials, a total of 4690 seeds were ingested by the six dabbling ducks, of which 634 (~13.52%) viable seeds were recovered from fecal samples, with percentages ranging from 3% to 30% depending on the dispersal and plant species (Table 2). Moreover, 94% of intact seeds were recovered within 12 h after feeding, and the remaining 5.8% were recovered within 12 h to 24 h. Only three seeds (<1%) were recovered after 24 h (Figure 1). The median seed retention time was 4 h or 5 h, except for *Polygonum orientale* (8 h) (Table 2). A total of 221 seeds germinated successfully, with the germination rate of all recovered seeds for each plant species ranging from 25% to 56% as compared with the control seeds (12~48%) (Figure 2 and Table 2). The quantities of germinated seeds in the experimental group were significantly higher than that in the control group for *Polygonum aviculare*, *Polygonum hydropiper*, *Polygonum orientale*, *Vallisneria natans* and *Carex cinerascen* (all *t* > 2.151; all *p* < 0.05). *Polygonum aviculare* seeds showed a higher retrieval rate. The average retention time of recovered seeds ranged from 4 h to 9 h depending on the seed and teal species. *Polygonum hydropiper* had maximum retention time for recovered and germinated seeds (30 h) along with the highest lignin (Table 1 and Table 2). Except for *Vallisneria natans*, the average retention time in the Baikal teals was longer than that in green-winged teals. Furthermore, the maximum retention time in Baikal teals was higher than or equal to that in green-winged teals.

Seed species demonstrated significant differences (ANOVA: *F*_7,72_ = 746.93, *p* < 0.001) in length and lignin (ANOVA: *F*_7,72_ = 185.56, *p* < 0.001). By contrast, Tukey tests revealed that 26 of 28 seed species pairs demonstrated significant differences in length (*p* < 0.001), and 23 of 28 SS pairs showed significant differences in lignin (*p* < 0.05).

Seed traits had significant effects on retention time. For average retention time (LMM1a), seed length and lignin had significant positive effects (*p* < 0.05). Seed retention time in green-winged teals was lower than that in Baikal teals but with no significant effect. Additionally, there was no significant interaction between teal species and seed length or lignin (Table 3). The model acquired a better fitting effect (with a marginal *R*^2^ of 51% and a conditional *R*^2^ of 63%) when the effect varied between dispersal individual and seed species. For maximum retention time (LMM1b), seed lignin had a significant positive effect (*p* < 0.05), whereas length had no significant effect. Moreover, the seed maximum retention time in Baikal teals was significantly higher than in green-winged teals, and we found no significant interaction between teal species and seed traits (Table 3). The value of marginal *R*^2^ (m*R*^2^) indicated that the fixed effects of LMM1b explained the 47% variance, and the remaining 8% was explained by the random factors.

The effect of seed lignin was significantly positive for the percentage of recovered seeds (GLMM2) (*p* = 0.001), but significant negative for the percentage of germinated seeds (GLMM3) (*p* < 0.05). Additionally, smaller seed showed a higher retrieval percentage, although the effect was not significant. The green-winged teal (with a smaller body) showed a significantly lower percentage of recovered seeds. Moreover, we found no significant interaction for seed retrieval within seed traits or between seed traits and teal species, and there was no interaction for seed germination. The fixed factors explained 68% of the variance, and random factors explained the remaining 12% in GLMM2. For GLMM3, 26% variance was explained by the fixed factors, and the random factors explained no variance (Table 3).

In GLMM4a, retention time squared (RT^2^) significantly negatively affected the recovered seeds per time interval (*p* = 0.001), whereas retention time (RT) had no significant effect. The teal species had a significant negative effect (*p* < 0.001), with the percentage of recovered seeds decreasing more quickly in the green-winged teals (Figure 1 and Table 3). Additionally, we analyzed the effect of retention time on germination in GLMM4b, with the results showing that both BS and RT^2^ had significant negative effects on the percentage of germinated seeds (*p* < 0.001), with no significant interaction observed between them (Table 3).

We then compared the effect of digestive tract on the germination of recovered seeds in the non-ingested control groups in GLMM5. The germination of seeds from gut passage was significantly higher than that in the controls (Figure 2 and Table 3). Moreover, seed species (SS) also played a significant role in the percentage of germinated seeds (*p* < 0.05), with the numbers of *Ranunculus polii* and *Euphrasia pectinata* significantly higher than those of other seed species. 

## 4. Discussion

These findings once again suggest that dabbling ducks (Anatinae) play a particularly important role in the seed dispersal of wetland plant species [14,19]. In this study, Baikal teals (*Anas formosa*) and green-winged teals (*Anas crecca*) were fed seeds, which then germinated (Figure 2, Table 2 and Table 3), indicating that waterbird-mediated endozoochory was feasible in the floodplain wetlands of the middle-lower Yangtze River floodplain. Additionally, green-winged teals are highly opportunistic and usually change their diet according to the season, with studies showing that more than 60% of plant seeds representative of a habitat can be found in their digestive system [37]. Therefore, it is necessary to study seed dispersal by green-winged teals. Moreover, further quantitative description of the seed dispersal effectiveness of wetland plants by ducks contributes to our understanding of wetland plant ecology and restoration [22].

The germination rate of recovered seeds ranged from 25% to 56%, which was similar to a previous study showing that fecal seeds germinated at a rate of 5% to 52% [38]; however, this differed considerably from another study that found a germination rate of 3% to 83% [28]. Additionally, the percentage of recovered seeds ranged from 3% to 30%, which is similar to previous findings [6,29]. The captive ducks in the feeding trials may have had short guts, which might have increased the number of recovered seeds and decreased the maximum retention time [39]. Moreover, the body size, diet, and habitat of the experimental subjects, as well as variations in the feeding trials, may also account for the differences between studies.

According to the shape of the curve (Figure 1), the germination rate of viable seeds from feces samples first increased and then decreased, which suggested that proper digestion may facilitate seed germination. However, if seeds are retained for a longer time in the gut, the digestive system can destroy the seed structure, thereby reducing the seed germination rate [28,30]. The difference in the rate of recovered seeds for each plant species between the Baikal teals and the green-winged teals was obvious, which may be due to their similarity in size and habits [40]. Moreover, the difference in the two species of ducks regarding the germination rate of each plant was not significant (*p* = 0.41; Table 3), suggesting that the vector species had little effect on recovered seed germination [30]. A previous study reported the median retention time of seeds in two wild duck species was 5 h, and the maximum retention time was 72–96 h [41], which was much higher than the 36 h observed in the present study. However, we found no intact seeds in the fecal samples after 24 h, indicating that the observation period selected in the present experiments was feasible [42].

Seed length has long been considered an important trait affecting seed dispersal. It has been suggested that smaller seeds have a higher retrieval and germination rate, as well as a shorter retention time [29]. The present results showed that smaller seeds passed through the digestive system more quickly, but there was no significant correlation between length and the percentage of recovered and geminated seeds (Table 3). Longer seeds may remain in the gizzard for a long time, whereas smaller seeds may pass through the gizzard more quickly [26]. Other studies showed that seed length had no effect on the retrieval rate of seeds from the guts of dabbling ducks [6,28]. The effects of seed length on dispersal are inconsistent, which may be due to other more important traits [14]. Lignin is one of the main components of plant cell walls, and its main function is to harden the cell walls by forming interlaced networks [43]. There has been little research on the effects of lignin on seed dispersal. In this study, we found a significant positive correlation between seed lignin and the average retention time and maximum retention time of recovered seeds (all *p* < 0.05; Table 3: GLMM2 and GLMM3,), which suggests that harder seeds with high lignin are tougher to digest and result in longer retention times in the guts [14]. Hard seed with high lignin may stay longer in the gizzard for mechanical digestion before moving into the guts, resulting in harder seeds surviving in the gut with a longer retention time [44], and thereby increasing the possibility of long-distance dispersal and the maximum distance [45,46]. Moreover, seed species with a higher lignin have a higher retrieval rate, indicating that the seed retention time affects the rate of recovered seeds. Based on observations during the feeding trial, the palatability of *E. pectinata* and *R. polii* seeds was high, whereas that of *Polygonum orientale*, *Polygonum hydropiper*, *Carex cinerascen*, and *Polygonum aviculare* seeds were poor. According to the data (Table 1), this suggests that the lignin was related to seed palatability. Seed traits can affect seed retrieval and germination, thereby affecting seed-dispersal distribution.

We found that disperser species had a significant influence on the maximum retention time of seeds in the gut. The maximum retention time of recovered seeds in the Baikal teal was generally higher than that of the green-winged teal (Table 3: LMM1b), likely to be due to the difference in body size. A previous study showed that the disperser species had a major influence on seed dispersal [47], and this study reached similar conclusions. The size difference of the carriers examined by Schupp et al. was large, although the size difference between the dabbling ducks in this study was smaller. The size of forest birds, not just waterbirds, may also affect the efficiency of seed dispersal [48].

Furthermore, seed retention time is an important parameter affecting the percentage of recovered seeds, which determines the distance of seed dispersal. In this feeding trial, 94% of the viable seeds were recovered within 12 h after feeding, which is similar to the results reported by Reynolds and Cumming [30]. Moreover, the present results showed that retention time squared had a significant negative effect on the number of recovered seeds. Another study indicated that a short retention time with a lower risk of digestion results in a higher number of recovered seeds [32]. Additionally, the present findings indicated that germination decreased along with a greater retention time, which was similar to the results of a previous study [28]. Furthermore, the present study showed that local seed dispersal by dabbling ducks was more frequent and effective than long-distance dispersal, whereas retention times of more than 48 h demonstrated the possibility of long-distance dispersal [41]. Altogether, the present findings demonstrated that endozoochory by dabbling ducks represents a widespread and effective dispersal mechanism among wetland plants.

This study has limitations. First, we did not investigate possible differences in body weight, diet, and habitat among the disperser species, which may limit the conclusions as presented. Second, there may exist differences in results between the present study and those previously reported, especially with respect to seed length; therefore, further studies are required to evaluate the impact of such differences. Finally, we did not evaluate the effect of nutrients in feces on seed germination; therefore, this should be examined in future research.

## 5. Conclusions

This study elucidated the influence of seed length and lignin on endozoochory parameters by dabbling ducks and compared variations among different disperser species to a certain extent. We found that seed length had a significant effect on average retention time, whereas there was no correlation with the percentage of recovered and germinated seeds. Additionally, seeds with higher lignin were tough to digest resulting in longer retention times, along with higher retrieval and lower germination rates. Moreover, the percentage of germinated seeds was significantly higher in both Baikal teals and green-winged teals relative to non-ingested controls. Seed retention time is a key parameter affecting the seed retrieval from the guts and was affected by seed traits and disperser species. Most seeds were recovered within 12 h, indicating the importance of local seed dispersal. Although few seeds last in the gut for long periods of time, the findings demonstrated the possibility of long-distance dispersal.

## Figures and Tables

**Table 1:** Measured mean values of the seed traits (±SE) of eight plant species in this study.

Seed Species	Family	Mass (mg)	Length (mm)	Lignin (mg/g)
*Polygonum aviculare*	Polygonaceae	0.50 ± 0.00	0.91 ± 0.01	10.03 ± 0.04
*Polygonum hydropiper*	Polygonaceae	1.20 ± 0.01	1.66 ± 0.02	10.33 ± 0.04
*Polygonum orientale*	Polygonaceae	7.01 ± 0.07	2.98 ± 0.03	9.55 ± 0.09
*Rumex dentatus*	Polygonaceae	0.51 ± 0.00	0.94 ± 0.04	8.73 ± 0.17
*Euphrasia pectinata*	Orobanchaceae	3.36 ± 0.07	1.88 ± 0.03	6.73 ± 0.06
*Vallisneria natans*	Hydrocharitaceae	0.14 ± 0.00	1.86 ± 0.02	9.51 ± 0.13
*Carex cinerasce*	Cyperaceae	1.14 ± 0.00	1.19 ± 0.01	10.11 ± 0.14
*Ranunculus polii*	Ranunculaceae	1.21 ± 0.01	2.18 ± 0.02	6.87 ± 0.08

**Table 2:** Average (mean ± SE), median (Med) and maximum (Max) retention time (h) for recovered seed from the green-winged teals (GWT) and the Baikal teals (BT). The maximum retention time for seed germination (Gmax). The percentage of recovered and germinated seeds per plant species from the GWT, BT and control groups.

Seed Species	Retention Time								Retrievability%		Germination%		
	GWT				BT				GWT	BT	GWT	BT	Control
	Mean	Med	Max	G_max_	Mean	Med	Max	G_max_					
*P. avi*	4.71 ± 0.33	4	24	24	5.51 ± 0.95	4	24	24	24 ± 1	30 ± 6	25 ± 2	25 ± 0	16 ± 7
*P. hyd*	6.99 ± 0.36	4	30	30	7.94 ± 0.25	6	30	20	10 ± 1	24 ± 4	41 ± 2	28 ± 2	26 ± 7
*P. ori*	8.17 ± 0.25	8	16	16	8.33 ± 0.83	8	24	20	9 ± 1	19 ± 1	46 ± 2	31 ± 3	26 ± 6
*R. den*	4.43 ± 0.30	4	10	8	4.84 ± 0.19	4	10	8	8 ± 1	16 ± 1	40 ± 6	37 ± 2	22 ± 9
*E. pec*	4.00 ± 0.69	4	10	10	6.32 ± 1.15	5	16	16	4 ± 1	8 ± 2	50 ± 6	56 ± 9	48 ± 9
*V. nat*	5.70 ± 0.15	5	16	16	4.71 ± 0.27	4	20	8	7 ± 2	12 ± 2	55 ± 10	32 ± 4	12 ± 5
*C. cin*	5.36 ± 0.32	4	20	20	6.69 ± 0.31	5	24	20	10 ± 2	20 ± 6	39 ± 6	37 ± 7	22 ± 5
*R. pol*	5.00 ± 0.19	4	10	10	6.10 ± 0.24	4	16	16	3 ± 0.3	14 ± 4	39 ± 6	52 ± 4	36 ± 12

*P. avi: Polygonum aviculare, P. hyd: Polygonum hydropiper, P. ori: Polygonum orientale, R. den: Rumex dentatus, E. pec: Euphrasia pectinate, V. nat: Vallisneria natans, C. cin: Carex cinerascen, R. pol: Ranunculus polii*.

**Table 3:** Summary of averaged models for the average (T_ave_) and maximum (T_max_) retention time of recovered seeds, for the percentage of recovered (Retrieval) and germinated seeds (Germination), for the retention time interval on recovered and germinated seeds, and for treatment (TT) according to alternation of GLMMs (see Table S1), including parameter estimates (*β*), standard errors (± SE) and confidence intervals, as well as the *R*^2^ of the top model represented the model fitting effect. The confidence intervals (CIs) were in bold if they did not overlap zero, indicating significant variables. The *p*-value explained the significance level of the variables. * refers to interaction of fixed factors.

**LMM1a**	**T_ave_**	**CIs**				**LMM1b**	**T_max_**	**CIs**			
Variables	*β* ± SE	2.5%	97.5%	*p*-value	*R* ^2^	Variables	*β* ± SE	2.5%	97.5%	*p*-value	*R* ^2^
(Intercept)	1.79 ± 0.06	**1.67**	**1.91**	<0.001		(Intercept)	2.66 ± 0.09	**2.49**	**2.83**	<0.001	
BS(GWT)	−0.13 ± 0.07	−0.27	0.02	0.088	**m*R*^2^**	BS(GWT)	−0.24 ± 0.10	**−0.44**	**−0.04**	0.02	**m*R*^2^**
SL	0.15 ± 0.04	**0.08**	**0.23**	<0.001	0.514	SLI	0.27 ± 0.09	**0.09**	**0.46**	0.004	0.469
SLI	0.11 ± 0.04	**0.02**	**0.20**	0.015		BS(GWT)*SLI	0.18 ± 0.10	−0.02	0.38	0.075	
BS(GWT)*SLI	0.09 ± 0.05	−0.01	0.19	0.062	**c*R*^2^**	SL	0.10 ± 0.07	−0.04	0.23	0.152	**c*R*^2^**
BS(GWT)*SL	0.06 ± 0.05	−0.04	0.15	0.257	0.632						0.554
**GLMM2**	**Retrieval**	**CIs**				**GLMM3**	**Germination**	**CIs**			
Variables	*β* ± SE	2.5%	97.5%	*p*-value	*R* ^2^	Variables	*β* ± SE	2.5%	97.5%	*p*-value	*R* ^2^
(Intercept)	−1.82 ± 0.10	**−2.02**	**−1.62**	<0.001		(Intercept)	−1.01 ± 0.09	**−1.19**	**−0.84**	<0.001	
BS(GWT)	−0.68 ± 0.10	**−0.87**	**−0.49**	<0.001	**m*R*^2^**	SLI	−0.22 ± 0.09	**−0.40**	**−0.04**	0.010	**m*R*^2^**
SLI	0.35 ± 0.11	**0.13**	**0.56**	0.001	0.679	BS(GWT)	0.14 ±0.16	−0.19	0.47	0.410	0.216
BS(GWT)* SLI	0.18 ± 0.11	−0.04	0.40	0.110		SL	0.06 ± 0.08	−0.10	0.22	0.460	
SL	−0.03 ± 0.11	−0.26	0.18	0.730	**c*R*^2^**						**c*R*^2^**
BS(GWT)* SL	−0.17 ± 0.09	−0.35	0.01	0.070	0.802						0.216
SL* SLI	−0.24 ± 0.16	−0.57	0.09	0.150							
**GLMM4a**	**RT|Retrieval**	**CIs**				**GLMM4b**	**RT|Germination**	**CIs**			
Variables	*β* ± SE	2.5%	97.5%	*p*-value	*R* ^2^	Variables	*β* ± SE	2.5%	97.5%	*p*-value	*R* ^2^
(Intercept)	−3.53 ± 0.07	**−3.67**	**−3.39**	<0.001	**m*R*^2^**	(Intercept)	−3.57 ± 0.10	**−3.76**	**−3.35**	<0.001	**m*R*^2^**
BS(GWT)	−0.65 ± 0.11	**−0.87**	**−0.43**	<0.001	0.913	BS(GWT)	−0.51 ± 0.15	**−0.80**	**−0.22**	<0.001	0.870
RT	0.45 ±0.24	−0.04	0.93	0.071		RT^2^	−0.70 ± 0.10	**−0.89**	**−0.51**	<0.001	
RT^2^	−0.99 ± 0.28	**−1.56**	**−0.42**	0.001	**c*R*^2^**						**c*R*^2^**
BS(GWT)*RT	−0.23 ± 0.16	−0.56	0.09	0.161	0.958						0.870
**GLMM5**	**TT**	**CIs**									
Variables	*β* ± SE	2.5%	97.5%	*p*-value	*R* ^2^						
(Intercept)	−1.68 ± 0.27	**−2.20**	**−1.15**	<0.001							
SS (*R. den*)	0.36 ± 0.25	−0.13	0.86	0.144	**m*R*^2^**						
SS (*P. ori*)	0.39 ± 0.24	−0.09	0.87	0.110	0.257						
SS (*V. nat*)	0.19 ± 0.27	−0.35	0.73	0.486							
SS (*R. pol*)	0.73 ± 0.25	**0.24**	**1.22**	0.003	**c*R*^2^**						
SS (*P. hyd*)	0.30 ± 0.24	−0.17	0.77	0.211	0.257						
SS (*C. cin*)	0.30 ± 0.24	−0.17	0.78	0.214							
SS (*E. pec*)	0.95 ± 0.25	**0.45**	**1.45**	<0.001							
TT (ingested)	0.38 ± 0.14	**0.09**	**0.67**	0.011							

BS: bird species, GWT: green-winged teal. BT: Baikal teals, BS(BT) is the reference category for all GLMMs, but GLMM5 where it is TT (control). SL: seed length, SLI: seed lignin, SS: seed species. *R. den*: *Rumex dentatus*, *P. ori*: *Polygonum orientale*, *V. nat*: *Vallisneria natans*, *R. pol*: *Ranunculus polii*, *P. hyd*: *Polygonum hydropiper*, *C. cin*: *Carex cinerascen*, *E. pec*: *Euphrasia pectinate*, *P. avi*: *Polygonum aviculare. SS (P. avi)* was the reference category for GLMM5. RT: retention time, RT^2^: retention time squared.

## Data Availability

Not applicable.

## Supplementary Materials

The following supporting information can be downloaded at: https://www.mdpi.com/article/10.3390/biology11050629/s1, Table S1: Summary of selected generalized linear mixed models (GLMMs) for retention time, retrievability and germination and treatment of seeds.
