# Supplementary material for: Effect of Seed Traits and Waterbird Species on the Dispersal Effectiveness of Wetland Plants
Source: Biology (Basel). 2022 Apr 20;11(5):629. doi: 10.3390/biology11050629 (PMC9137643; doi:10.3390/biology11050629)
Supplement: Supplementary file 1 [file biology-11-00629-s001.zip › biology-1669876-supplementary.pdf]

biology MDPI

Supplementary Materials

Effect of Seed Traits and Waterbird Species on the Dispersal Effectiveness of Wetland Plants

Shenghong Nie, Lizhi Zhou and Wenbin Xu

Table S1. Summary of generalized linear mixed models (GLMMs) selection for investigating the effects of dispersal Scheme 10. and the Null model with only the constant and random fators. K is the number of estimated parameters, Wt the weight per model and LogLik is the -2log-likelihood output for each model. Each model has fixed and random effects.

LMM1a: Average retention time (T_{ave}); Random effects = (1|ID) + (1|SS)

Fixed Effects	K	AIC_c	ΔAIC_c	Wt	LogLik
BS + SL + SLI + BS*SLI	8	-8.74	0.00	0.22	14.22
SL + SLI	6	-8.60	0.14	0.20	11.32
BS + SL + SLI	7	-8.34	0.40	0.18	12.57
BS + SL + SLI + BS*SL +BS*SLI	9	-7.04	1.70	0.09	14.89
SL + SLI + SL*SLI	7	-6.13	2.61	0.06	11.47
BS + SL + SLI + BS*SLI +SL*SLI	9	-5.98	2.75	0.06	14.36
BS + SL + SLI + BS*SL	8	-5.79	2.95	0.05	12.74
BS + SL + SLI +SL*SLI	8	-5.74	3.00	0.05	12.71
BS + SL + SLI + BS*SL + BS*SLI + SL*SLI	10	-4.12	4.62	0.02	15.03
BS + SL + SLI + BS*SL + SL*SLI	9	-3.03	5.71	0.01	12.88
SL	5	-2.99	5.75	0.01	7.21
BS + SL	6	-2.71	6.03	0.01	8.38
Null	4	-1.78	6.96	0.00	5.35

BS: dispersal species, SL: seed length, SLI: seed lignin, * refers to interaction of fixed factors
ID: dispersal individual, SS: seed species

LMM1b: Maximum retention time (T_{max}); Random effects = (1|ID) + (1|SS)

Fixed Effects	K	AIC_c	ΔAIC_c	Wt	LogLik
BS + SLI + BS*SL I	7	55.99	0.00	0.19	-19.60
BS + SLI	6	56.48	0.49	0.15	-21.22
BS + SL + SLI + BS*SLI	8	56.95	0.96	0.12	-18.63
BS + SL + SLI	7	57.31	1.32	0.10	-20.25
BS + SL + SLI + BS*SLI + SL*SLI	9	58.01	2.02	0.07	-17.63
SLI	5	58.20	2.21	0.06	-23.39
BS + SL + SLI + SL*SLI	8	58.21	2.21	0.06	-19.26
BS + SL + SLI + BS*SL	8	58.46	2.47	0.06	-19.39
SL + SLI	6	58.96	2.97	0.04	-22.46
BS + SL + SLI + BS*SL + BS*SLI	9	59.19	3.20	0.04	-18.23
BS + SL + SLI + BS*SL + SL*SLI	9	59.52	3.53	0.03	-18.39

SL + SLI + SL*SLI	7	59.80	3.81	0.03	-21.50
BS + SL + SLI + BS*SL + BS*SLI + SL*SLI	10	60.40	4.41	0.02	17.23
BS	5	63.53	7.54	0.00	-26.05
Null	4	65.15	9.16	0.00	-28.11

BS: dispersal species, SL: seed length, SLI: seed lignin, * refers to interaction of fixed factors
ID: dispersal individual, SS: seed species

GLMM2: Retrieval; Random effects = (1|ID) + (1|SS)

Fixed Effects	K	AIC_c	ΔAIC_c	Wt	LogLik
BS + SLI + BS*SLI	6	279.14	0.00	0.21	-132.54
BS + SLI	5	279.25	0.12	0.19	-133.91
BS + SL + SLI + BS*SL	7	280.07	0.94	0.13	-131.64
BS + SL + SLI	6	280.97	1.83	0.08	-133.46
BS + SL + SLI + BS*SLI	7	280.98	1.85	0.08	-132.09
BS+ SL + SLI + BS*SL + SL*SLI	8	281.00	1.86	0.08	-130.65
BS + SL + BS*SL + BS*SLI	8	281.59	2.46	0.06	-130.95
BS + SL + SLI + SL*SLI	7	281.79	2.66	0.05	-132.50
BS + SL + SLI + BS*SLI + SL*SLI	8	282.00	2.87	0.05	-131.16
BS + SL + SLI + BS*SL + BS*SLI + SL*SLI	9	282.77	3.63	0.03	-130.06
BS	4	285.29	6.16	0.01	-138.18
BS + SL + BS*SL	6	285.46	6.34	0.01	-135.71
BS + SL	5	286.42	7.28	0.01	-137.50
Null	3	298.40	19.26	0.00	-145.93

BS: dispersal species, SL: seed length, SLI: seed lignin, * refers to interaction of fixed factors
ID: dispersal individual, SS: seed species

GLMM3: Germinated; Random effects = (1|ID) + (1|SS)

Fixed Effects	K	AIC_c	ΔAIC_c	Wt	LogLik
SLI	4	159.41	0.00	0.37	-75.24
BS + SLI	5	161.20	1.79	0.15	-74.88
SL + SLI	5	161.34	1.93	0.14	-74.95
BS + SL + SLI	6	163.19	3.78	0.06	-74.57
BS + SLI + BS*SLI	6	163.26	3.86	0.05	-74.61
Null	3	163.46	4.06	0.05	-78.46

BS: dispersal species, SL: seed length, SLI: seed lignin, * refers to interaction of fixed factors
ID: dispersal individual, SS: seed species

GLMM4a: Recovered within time interval (RT|Retrieval); Random effects = (RT|ID)

Fixed Effects	K	AIC_c	ΔAIC_c	Wt	LogLik
BS + RT + RT^2	7	225.71	0.00	0.33	-103.85
BS + RT^2	6	225.91	0.21	0.30	-105.51
BS + RT + RT^2 + BS*RT	8	227.14	1.44	0.16	-102.91
BS + RT + RT^2 + BS*RT^2	8	228.30	2.59	0.09	-103.48
BS + RT^2 + BS*RT^2	7	228.59	2.88	0.08	-105.30
BS + RT + RT^2 + BS*RT + BS*RT^2	9	229.46	3.76	0.05	-102.27
Null	4	271.73	46.02	0.00	-131.22

BS: dispersal species, RT: retention time, RT^2: retention time squared, * refers to interaction of fixed factors
RT|ID: dispersal individual within time interval

GLMM4b: Germinated within time interval (RT|Germination); Random effects = (RT|ID)

Fixed Effects	K	AIC_c	ΔAIC_c	Wt	LogLik
BS + RT^2	6	167.76	0.00	0.50	-76.43
BS + RT^2+ BS*RT^2	7	169.84	2.09	0.17	-75.92
BS + RT + RT^2	7	170.49	2.73	0.13	-76.25
BS + RT + RT^2 + BS*RT	8	172.23	4.47	0.05	-75.45
BS + RT + RT^2 + BS*RT^2	8	172.75	4.99	0.04	-75.71
BS + RT	6	172.84	5.08	0.04	-78.97
RT^2	5	173.38	5.62	0.03	-80.69
BS + RT + BS*RT	7	174.62	6.87	0.02	-78.31
BS + RT + RT^2 + BS*RT + BS*RT^2	9	175.18	7.42	0.01	-75.13
RT + RT^2	6	175.93	8.17	0.01	-80.51
Null	4	190.96	23.21	0.00	-90.84

BS: dispersal species, RT: retention time, RT^2: retention time squared, * refers to interaction of fixed factors
RT|ID: dispersal individual within time interval

GLMM5: Treatment (TT); Random effects = (1|ID)

Fixed Effects	K	AIC_c	ΔAIC_c	Wt	LogLik
TT + SS	10	292.10	0.00	0.66	-134.62
TT	3	294.07	1.96	0.25	-143.89
Null	2	296.73	4.63	0.07	-146.29

SS: seed species, TT: treatment, * refers to interaction of fixed factors
ID: dispersal individual
